# Co-expression and co-responses: within and beyond transcription

**DOI:** 10.3389/fpls.2012.00248

**Published:** 2012-11-08

**Authors:** Takayuki Tohge, Alisdair R. Fernie

**Affiliations:** Max-Planck-Institute of Molecular Plant PhysiologyPotsdam-Golm, Germany

**Keywords:** gene annotation, network analysis, gene expression, plant metabolism, correlation analysis

## Abstract

Whole genome sequencing, the relative ease of transcript profiling by the use of microarrays and latterly RNA sequencing approaches have facilitated the capture of vast amounts of transcript data. However, despite the enormous progress made in gene annotation a substantial proportion of genes remain to be annotated at the functional level. Considerable progress has, however, been made by searching for transcriptional coordination between genes of known function and non-annotated genes on the premise that such co-expressed genes tend to be functionally related. Here we review progress made following this approach as well as its expansion to include phenotypic information from other levels of cellular organization such as proteomic and metabolomic data as well as physiological and developmental phenotypes.

## INTRODUCTION

Despite the laudable aim of the Arabidopsis 2010 project we remain a long way from knowing the function of every gene of this plant, notwithstanding unprecedented research effort with recent estimates suggesting in the region of 50% of genes are functionally annotated by gene homology and between 10 and 15% have an experimentally verified biological function (Saito et al., 2008; Tohge and Fernie, 2010; Mutwil et al., 2011). The simplicity of homology searches means that at least for dicots the number of genes annotated by homology in rice and soybean or in the more recently published maize (Schnable et al., 2009), poplar (Tuskan et al., 2006), or tomato (Tomato Genome Consortium, 2012) genomes remains reasonable. However, the proportion of genes for which function has been verified experimentally is, at least in most of these species, negligible rendering predictive gene annotation and subsequent validation thereof a vital task for genomics both in model and crop species.

The development and widespread adoption of unbiased RNA sequencing (RNAseq) approaches by plant researchers (Bao et al., 2011; Matas et al., 2011; Hamilton and Buell, 2012; Lohse et al., 2012) effectively increases the scale of this task since it circumvents the need for in depth *a priori* knowledge that was a pre-requisite for microarray hybridizations. Despite the fact that we have as yet not reached satisfactory levels of gene annotation several approaches – all of which are based on a common principal – have recently greatly facilitated gene annotation. This is particularly in the case of pathways under strict transcriptional regulation such as cell wall associated genes and those involved in the various pathways of secondary metabolism as well as leading to the classification of process-associated gene including those linked to cold stress and jasmonate signaling, operon-like genes and seed germination (Hannah et al., 2005; McGrath et al., 2005; Tohge et al., 2005; Saito et al., 2008; Srinivasasainagendra et al., 2008; Mutwil et al., 2009; Obayashi et al., 2009; Usadel et al., 2009; Ogata et al., 2010; Tohge and Fernie, 2010; Bassel et al., 2011; Wada et al., 2012). These approaches are based on the guilt-by-association approach which assumes that if transcript levels of a gene of unknown function co-respond tightly with those of a gene of known function then it is highly likely that the gene of unknown function plays a role in the same biological process as the known gene. Whilst by no means foolproof, providing a number of considerations and caveats are taken into account, as pointed out in an excellent review by many of the leading investigators in the field (Usadel et al., 2009), then this strategy can prove very powerful. In this mini-review we detail (i) how such approaches have been utilized in a “stand-alone” fashion to successfully predict gene function in Arabidopsis, (ii) how such approaches can be translated for gene functional prediction in crop species for which suitable transcriptomic datasets are publically available, and finally (iii) how other phenotypic data can be incorporated into such studies to support successful gene annotation.

## PREDICTION OF THE FUNCTION OF ARABIDOPSIS GENES

In spite of the clear advantage of biological co-expression network approaches based on gene expression, protein interaction, and genetic interactions for microorganisms such as yeast (see an example, Zhang et al., 2005), co-expression network approaches in plant research have largely been developed solely on the basis of microarray data. This has revealed clear correlations between genes in multiple biosynthetic pathways (Tohge et al., 2007; Movahedi et al., 2011; Mutwil et al., 2011). In addition, *Arabidopsis thaliana* is currently the most useful model plant for integrative analysis due to the availability of several resources such as knockout mutants, cDNA library, tag counts of ESTs, microarray, data and metabolite profiling data. Furthermore, several co-expression gene network analyses and integrative analysis with metabolite profiles have been used to understand the transcriptional correlation networks and discover novel gene functions in this species (Noji et al., 2006; Saito et al., 2008; Mao et al., 2009; Tohge and Fernie, 2010; Mutwil et al., 2011). For this purpose, several web-based co-expression applications, for example ATTED-II (Obayashi et al., 2009, 2011), AraNet (Hwang et al., 2011), Expression Angler of the Bio-Array Resource (BAR; Toufighi et al., 2005), CressExpress (Srinivasasainagendra et al., 2008), CSB.DB (Steinhauser et al., 2004), KappaViewer (Sakurai et al., 2011), GeneCAT (Mutwil et al., 2008), Genevestigator (Zimmermann et al., 2004), OryzaExpress (Hamada et al., 2011), and VirtualPlant (Katari et al., 2010) have been developed (**Table 1**).

**Table 1:** Co-expression databases presented in this article.

**Species**	**Database**	**URL**
**Cross species**
	PlaNet	http://aranet.mpimp-golm.mpg.de
***Arabidopsis thaliana***
	ATTED-II	http://atted.jp/
	AraNet	http://www.functionalnet.org/aranet/
	BAR	http://bar.utoronto.ca/welcome.htm
	COP	http://webs2.kazusa.or.jp/kagiana/cop
	CORNet	http://bioinformatics.psb.ugent.be/cornet
	CSB.DB	http://csbdb.mpimp-golm.mpg.de/csbdb/dbcor/ath.html
	CressExpress	http://www.cressexpress.org/index
	GeneCAT	http://genecat.mpg.de/cgi-bin/Ainitiator.py
	GeneVestigator	https://www.genevestigator.com/gv/plant.jsp
	KappaViewer4	http://kpv.kazusa.or.jp/en/
	SeedNet	http://bree.cs.nott.ac.uk/arabidopsis/
***Oryza sativa***
	OryzaExpress	http://bioinf.mind.meiji.ac.jp/Rice_network_public/script/index.html
	ATTED-II	http://atted.jp/
***Populus trichocarpa***
	COP	http://webs2.kazusa.or.jp/kagiana/cop/

One of the best examples of co-expression analysis in Arabidopsis is cellulose synthase (CESA) genes in the secondary cell wall metabolism (Brown et al., 2005; Persson et al., 2005b), and the primary wall hemicellulose xyloglucan (Cocuron et al., 2007). These studies used the three major secondary wall CESA genes as the baits to construct networks and find novel functional genes displaying similar expression patterns. The CESA gene network has been in several publications (Persson et al., 2005a; Mutwil et al., 2008, 2009, 2011; Ruprecht et al., 2011). A second successful example of the co-expression approach is that of plant secondary metabolism, since this is of the directly regulated at the transcriptional level by a range of different transcription factors including the MYB transcription factors. Since a framework of flavonoid co-expression network was constructed for identify the flavonol-3′-*O*-methyltransferase (AtOMT1; Tohge et al., 2007), such co-expression network approaches have been expanded to find other flavonoid biosynthetic genes such as flavonol-7-*O*-rhamnosyltransferase (At1g06000) and flavonol-3-*O*-arabinosyltrasnferase (At5g17030; Yonekura-Sakakibara et al., 2007, 2008; Tohge and Fernie, 2010). In addition, this approach was also utilized in the identification of glucosinolate MYB regulators (AtMYB28 and AtMYB29; Hirai et al., 2007), monolignol transporter (AtABCG29) involved in lignin biosynthesis (Alejandro et al., 2012) and novel signaling related candidate genes and transporters following the exposure of Arabidopsis to UV-B (Tohge et al., 2011a).

In addition to its utility in understanding the regulation of individual metabolic pathways or even metabolic networks co-expression analysis has also been applied at a much broader level to look at tissue-specific transcriptional networks (Song et al., 2010) and at diverse biological processes including seed germination and dark-induced senescence (Araújo et al., 2011; Bassel et al., 2011). Studying gene sharing networks of Arabidopsis and rice Song et al. (2010) discovered that tissues or cell types from the same organ system tend to group together to form network modules. The operon-like clusters in Arabidopsis using genome-based co-expression network analysis has been found (Wada et al., 2012). Furthermore, plant tissues in consecutive developmental stages or sharing physiological functions are highly connected. Extending their comparisons to mouse and human gene expression data they were able to observe common principles of gene-sharing across the species and hypothesize that gene sharing evolved as a fundamental organizing feature of gene expression in eukaryotes. The co-expression approach was also successfully applied to microarray data across the entire seed germination process (Bassel et al., 2011). The output, which the authors termed SeedNet (http://vseed.nottingham.ac.uk), facilitated the definition of two state-dependent interactions associated with either dormancy or germination with an intermediate transition region between the two being characterized by an enrichment of genes involved in cellular phase transitions. Moreover, the dormancy region of the co-expression network was strongly associated to abiotic stress response genes. The combined findings were thus taken to suggest that seed dormancy is an adaptive trait that arose evolutionarily late and evolved by coopting existing biosynthetic pathways regulating cellular phase transitions and abiotic stress response genes. During dark-induced senescence there is a dramatic switch from respiration of sugars to respiration of protein which is underpinned by dramatic transcriptional reprogramming of metabolism (Araújo et al., 2010 Araújo et al., 2011), including the degradation of lysine and branched chain amino acids by as yet undefined pathways. In this case the co-expression response was able to provide a high number of candidate genes involved in this process (Araújo et al., 2011), however these remain to be functionally verified.

## PREDICTION OF THE FUNCTION OF CROP GENES: WITHIN SPECIES COMPARISONS

Although considerably fewer microarray experiments have been reported for crop species, with the possible exceptions of rice, several examples exist of the power of the approach in stand-alone network analyses for rice and tomato (Ficklin et al., 2010; Ozaki et al., 2010; Rohrmann et al., 2011; Sakurai et al., 2011; Fukushima et al., 2012). We will here shortly review these studies and highlight the important knowledge inference for studies in tomato and the grasses. In tomato the most comprehensive study was that performed by Fukushima et al. (2012) who constructed co-response networks from 327 tomato Affymetrix arrays. Although this dataset was substantially smaller than that regularly used for Arabidopsis a number of important conclusion could be drawn including biologically relevant co-expression networks including DNA endoreduplication, response to cold, jasmonate-associated metabolic processes, and the ubiquitous photosynthetic gene cluster. The study also revealed that duplicated genes often displayed differential co-expression when tissue-type was studied a fact highlighted by genes of lycopene and flavonoid biosynthesis (Fukushima et al., 2012). In two more targeted analyses co-expression analysis was also linked to metabolite levels in tomato fruit (Rohrmann et al., 2011; Lee et al., 2012), however, we will return to these studies later when discussing *layering in other phenotypes to aid annotation strategies.*

In addition to these recent studies in tomato there have also been studies in barley (*Hordeum vulgare*; Faccioli et al., 2005; Mochida et al., 2011; Tohge et al., 2011b), wheat (Manickavelu et al., 2012), rice (Fukushima et al., 2009; Lee et al., 2009; Ficklin et al., 2010; Childs et al., 2011; Hamada et al., 2011), maize (Ficklin and Feltus, 2011), poplar (*Populus* spp.; Ogata et al., 2010), and tobacco (*Nicotiana tabacum*; Edwards et al., 2010). Studies in rice revealed that gene co-expression analysis facilitated elucidation of gene function. With the study of Ficklin et al. (2010) returning 45 co-expressed gene modules and 76 cofunctional gene clusters some of which were enriched for previously characterized mutant phenotypes thus providing strong hints toward molecular functions of unknown genes within the clusters with similar outcomes being achieved for the other species mentioned above.

## PREDICTION OF THE FUNCTION OF CROP GENES: BETWEEN SPECIES COMPARISONS

Whilst the above described studies show that there is considerable benefit from co-expression analysis in species such as tomato for which genome scale microarray platforms do not yet exist another approach that has been demonstrated to be highly powerful is combining comparisons of gene cluster networks and sequence homology as a method of assigning gene function and was recently published under the acronym PlaNet (Mutwil et al., 2011). PlaNet builds on the concept first published in 2008 by the same group which already described the search for barley gene orthologs of annotated Arabidopsis genes (Mutwil et al., 2008). PlaNet extended this to include the crop species barley, medicago, poplar, rice, soybean, and wheat, and used a comparative network algorithm to estimate similarities between network structures. The algorithm was exemplified using the canonical the photosystem I reaction center (*PSA-D*) family gene-related networks as well as those related to chalcone synthase suggesting that the rapid transfer of knowledge between species will be possible. That this is so was recently also demonstrated by the same group in a study of secondary wall cellulose biosynthesis (Ruprecht et al., 2011). In this study, the authors compared co-expressed gene vicinity networks of primary and secondary wall CESAs in all species housed in PlaNet to identify those genes consistently co-regulated with cellulose biosynthesis. In addition to the expected polysaccharide acting enzymes, they also found many gene families associated with cytoskeleton, signaling, transcriptional regulation, oxidation, and protein degradation. Based on these analyses, they selected and biochemically analyzed T-DNA insertion lines corresponding to approximately 20 genes from gene families that re-occur in the co-expressed gene vicinity networks of secondary wall CESAs across the seven species. One of the mutants, corresponding to a pinoresinol reductase gene, was subsequently characterized as displaying disturbed xylem morphology and containing lower levels of lignin than the wild-type.

The very same seven species used for the PlaNet study were used in an independent study to generate a pipeline within the BAR software suite (Toufighi et al., 2005) to rank ortholog predictions based on sequence and expression profile similarity with the best fitting on this criteria being defined as the *expressolog* (Patel et al., 2012). Interestingly, global analyses revealed that orthologs with the highest sequence similarity do not necessarily exhibit the highest expression pattern similarity. Moreover, other putative orthologs show highly distinct expression patterns suggesting they may need re-annotating or at best to be given a more specific annotation. A similar comprehensive comparison between maize and rice was additionally recently carried out using the IsoRank tool (Ficklin and Feltus, 2011). It thus appears likely that both these tools as well as PlaNet will likely greatly aid translational efforts to translate the huge knowledge we have gained from Arabidopsis studies into crop species.

## LAYERING IN OTHER PHENOTYPES TO AID ANNOTATION STRATEGIES

The above examples have by and large only relied on data from transcript profiling and have neither harnessed information derived from other molecular approaches, such as proteomics and metabolomics, nor indeed of end-phenotypes such as total yield and harvest indexes. Several recent studies have however incorporated such data collected in order to complement transcriptomic efforts of gene functional annotation (Hirai et al., 2007; Horan et al., 2008; Yonekura-Sakakibara et al., 2008; Sulpice et al., 2009; Allen et al., 2010; Tohge and Fernie, 2010; Araujo et al., 2011; Rohrmann et al., 2011; Tohge et al., 2011a,2011b). Returning to the tomato examples mentioned above, in order to exploit the impact of tomato genetic diversity on carotenoids, Lee et al. (2012) used *Solanum pennellii* introgression lines as a source of defined natural variation and as a resource for the identification of candidate regulatory genes. For this purpose ripe fruits were analyzed for numerous fruit metabolites and transcriptome profiles generated using a 12,000 unigene oligoarray. Correlation analysis between carotenoid content and gene expression profiles revealed 953 carotenoid-correlated genes. A subnetwork analysis of carotenoid-correlated transcription narrowed this down to 38 candidates. One of which, *Solanum lycopersicum* ethylene response factor 6 (*SlERF6*), was subsequently functionally characterized revealing that it indeed influences carotenoid biosynthesis and additional ripening phenotypes. In a similar approach Rohrmann et al. (2011) developed a quantitative real-time PCR platform allowing accurate quantification of the expression level of approximately 1000 tomato transcription factors. In addition to utilizing this novel approach, they performed cDNA microarray analysis and metabolite profiling of primary and secondary metabolites using gas chromatography–mass spectrometry (GC–MS) and liquid chromatography–mass spectrometry (LC–MS), respectively. Applying these platforms to pericarp material harvested throughout fruit development and studying both wild-type *Solanum lycopersicum* cv. Ailsa Craig and the *hp1* (*high pigment*) mutant which is functionally deficient in the tomato homolog of the negative regulator of the light signal transduction gene UV-DAMAGED DNA BINDING PROTEIN 1 (*DDB1*) from Arabidopsis. They chose this particular mutant since it had previously been shown to harbor dramatic alterations in the content of several important fruit metabolites but relatively little impact on other ripening phenotypes. The combined dataset was extensively mined searching for co-responsive metabolites and transcription factors, and, where possible, the respective transcriptional expression network underlying this control. Two further studies in tomato merit discussion here. Mounet et al. (2009), used a combination of metabolite profiling and transcript profiling to identify candidate for the key factor of fruit composition and development. More recently Osorio et al. (2011), used a combination of transcriptomics, proteomics, and metabolomics alongside network computation to assess ripening across a range of classical ripening mutants and recently extended this analysis to compare ripening in tomato with that in pepper (Osorio et al., 2012).

Staying with the integration of transcriptomic, proteomic, and metabolomic data we recently combined data from all three platforms to infer function within the tonoplast proteome (Tohge et al., 2011b). In order to do so we performed metabolic profiling of both primary and secondary metabolites in highly purified vacuoles of barley or the protoplast preparations from which they were isolated. This gave us quantitative data on 59 primary metabolites for which we knew the exact chemical structure and some 200 secondary metabolites for which we had strong predicted chemical formulae. This data was then compared to the 88 tonoplast proteins reported for barley (Endler et al., 2006) and evaluating there co-expression using PlaNet. This strategy allowed us to putatively assign transport function for phenylpropanoids, flavonoids, storage proteins, and mugi-neic acid, as well as a potential transport system for phytosiderophores.

Proteomic data are also an important component of the interaction networks that form part of the CORNET tools (De Bodt et al., 2010, 2012) which combine co-expression analysis with protein–protein interaction searches. The latter is similar to other tools such as those in CressExpress, BAR, and VirtualPlant (Toufighi et al., 2005; Srinivasasainagendra et al., 2008; Katari et al., 2010), however, it presents microarray data with the corresponding meta-data including sample information, protein–protein interaction data, localization data, and functional information within a single central database. Developed CORNET 2.0 includes the majority of interaction databases, six different protein–protein interaction dataset, and three sets of regulatory interaction data, thereby providing with consistently updated data sets for versatile searches (De Bodt et al., 2012). The efficacy of computational classification to enrich potential protein–protein interactions to predict putative interactions of Arabidopsis membrane protein has been applied (Chen et al., 2012). This method is also an important to fill gaps to biological networks and suggest hypothetical process and genes involving signal transduction and transport.

## CONCLUSION

It is hopefully clearly apparent from this mini-review that co-expression analyses are a very powerful tool in gene annotation not only in model systems such as Arabidopsis and rice but also in less well characterized plant species. To date, it has found great utility in improving our understanding of pathways which are known to be regulated at the transcriptional level such as cell wall biosynthesis and various pathways of secondary metabolism, however, recent examples also demonstrate its utility in elucidating novel players in various developmental processes. The guilt-by-association response is clearly powerful even in stand-alone single species approaches. However, the increasing availability of data from multiple species and at multiple different levels of the cellular hierarchy will likely facilitate the adoption of integrative genomics approaches by many more laboratories in the near future. Even some 6-9 years ago the power of combining transcript and metabolite profiling for (candidate) gene discovery was demonstrated for non-sequenced species (Urbanczyk-Wochniak et al., 2003; Rischer et al., 2006). Recent developments in RNA sequencing (Schneeberger and Weigel, 2011), will likely render this considerably easier in the near future.

## Conflict of Interest Statement

The authors declare that the research was conducted in the absence of any commercial or financial relationships that could be construed as a potential conflict of interest.
